# Exploring Emotional Distress, Psychological Traits and Attitudes in Patients with Chronic Migraine Undergoing OnabotulinumtoxinA Prophylaxis versus Withdrawal Treatment

**DOI:** 10.3390/toxins12090577

**Published:** 2020-09-08

**Authors:** Valeria Donisi, Maria Angela Mazzi, Marialuisa Gandolfi, Giuseppe Deledda, Fabio Marchioretto, Simone Battista, Sara Poli, Matteo Giansante, Eleonora Geccherle, Cinzia Perlini, Nicola Smania, Lidia Del Piccolo

**Affiliations:** 1 Section of Clinical Psychology, Department of Neurosciences, Biomedicine and Movement Sciences, University of Verona, Piazzale L.A. Scuro, 10, 37134 Verona, Italy; valeria.donisi@univr.it (V.D.); mariangela.mazzi@univr.it (M.A.M.); cinzia.perlini@univr.it (C.P.); lidia.delpiccolo@univr.it (L.D.P.); 2 Department of Neurosciences, Biomedicine and Movement Sciences, Neuromotor and Cognitive Rehabilitation Research Centre (CRRNC), University of Verona, UOC Neurorehabilitation, AOUI Verona, Piazzale L.A. Scuro, 10, 37134 Verona, Italy; nicola.smania@univr.it; 3 Clinical Psychological Service, UO of Clinical Psychology, Scientific Institute for Research, Hospitalisation and Health Care (IRCCS), Sacro Cuore—Don Calabria, Negrar di Valpolicella, 37024 Verona, Italy; giuseppe.deledda@sacrocuore.it (G.D.); sara.poli@sacrocuore.it (S.P.); matteo.giansante@sacrocuore.it (M.G.); eleonora.geccherle@sacrocuore.it (E.G.); 4 Neurological Unit, Scientific Institute for Research, Hospitalisation and Health Care (IRCCS), Sacro Cuore—Don Calabria, Negrar di Valpolicella, 37024 Verona, Italy; fabio.marchioretto@sacrocuore.it; 5 Department of Neurosciences, Rehabilitation, Ophthalmology, Genetics, Maternal and Child Health, University of Genova, Campus of Savona, Via Magliotto, 2, 17100 Savona, Italy; simone.battista@edu.unige.it

**Keywords:** chronic migraine, coping strategies, headache-related disability, psychological factors, psychological distress, OnabotulinumtoxinA prophylactic treatment

## Abstract

This explorative cross-sectional study aims at exploring emotional distress, psychological profiles, and the attitude towards receiving psychological support in eighty-seven patients with chronic migraine (CM) undergoing OnabotulinumtoxinA prophylactic treatment (OBT-A, n = 40) or withdrawal treatment (WT, n = 47). The outcomes were explored through a specific battery of questionnaires. 25% of patients undergoing OBT-A and almost half of the patients undergoing WT reported psychological distress of at least moderate-severe level, respectively. Coping strategies, self-efficacy, and perceived social support were similar in the two groups. Patients undergoing OBT-A presented lower psychological inflexibility than patients undergoing WT. Predictors of higher psychological distress were low perceived social support by friends, low self-efficacy, and higher avoidance strategies. In both groups, most of the patients evaluated receiving psychological support to be useful (79%). The potential beneficial effects of OBT-A on the severity of symptoms and psychological distress might further support its role in the multidisciplinary management of patients with CM. Identifying patients with psychological vulnerabilities who may benefit from psychological support is relevant in patients with CM.

## 1. Introduction

Migraine is one of the major causes of disability worldwide in adults under-50s [1,2]. Chronic migraine (CM) represents the most severe and disabled portion of the migraine spectrum when compared with both non-migraine and episodic migraine (EM). It affects patients’ wellbeing and quality of life, with a negative impact on relationships, functioning, and personal roles [3,4,5,6,7,8,9]. CM is defined as at least 15 days of headache per month for three months or more. Only eight of these headache days per month need to meet migraine diagnostic criteria [10]. Different risk factors, including overuse of acute migraine medication (MOH), ineffective acute treatment, obesity, depression, and stressful life events have been related to migraine chronification, which occurs in about 2.5–3% of patients with EM [11,12,13]. The observation that about 26% of patients with CM might reverse into remission within two years of chronification [11] supports the importance of early identifying risks factors for chronification and adopting an integrated treatment to improve their management. A variety of psychological diagnoses, such as depression, anxiety, obsessive-compulsive, substance abuse, sleep problems, and sub-threshold psychological symptoms, have often resulted in an association with CM, as in other conditions leading to chronic pain [14,15,16,17]. For instance, the association between migraine and psychological distress is higher in CM than in EM, especially in patients with MOH. In this case, a high prevalence of psychopathological comorbidities has been reported [17,18,19,20,21]. Typically, depression and anxiety symptoms increase with headache frequency and are associated with higher levels of migraine-related disability [16,17,22,23,24,25]. However, in the clinical practice often psychological distress is not reported in the physician-patient encounter, due, for example, to fear of stigmatization or the tendency to suppress emotions [26,27,28,29].

The first-line management of CM is based on pharmacological treatment, according to the pathophysiology of the disturbance [11]. However, the bulk of literature is focused on improving the pharmacological outcome of this highly disabling disturbance through a multidisciplinary pathway of care [30,31,32,33,34,35,36,37]. Psychological assessment and treatment for migraine should be integrated within this multidisciplinary approach early, to enhance adherence to pharmacological and non-pharmacological treatments and improve pain management, as in other neurological conditions causing chronic pain, as suggested by the Italian Consensus Conference on Pain in Neurorehabilitation [38,39]. Several psychological constructs have been studied as potential mediators of psychological distress, perceived pain, migraine management and prognosis (e.g., locus of control, self-efficacy, social support, psychological flexibility, and coping strategies). These factors might be taken into account in order to understand variation in disability and quality of life in CM [11,16,34,35,40,41,42,43,44,45,46,47,48] within the conceptualization of migraine according to a bio-psycho-social model, where biological and psychosocial factors are strictly interconnected [47,49].

To the best of our knowledge, most studies on psychological factors focused on patients with CM and MOH, often during withdrawal treatment (WT). Only a few studies have explored psychological factors during specific prophylaxis treatment, such as that with OnabotulinumtoxinA prophylactic treatment (OBT-A). OnabotulinumtoxinA is an effective and well-tolerated treatment of CM [50], reducing the severity of symptoms in terms of headache and migraine days, cumulative hours of headache on headache days, and frequency of moderate/severe headache days. Therefore, a reduction of the burden of illness in adults with disabling chronic migraine has been reported [50]. OBT-A has been associated with a reduction in symptoms of depression and anxiety in patients with CM [51,52]. Although other studies reported no significant OBT-A benefit on psychological disturbances [53,54], an antidepressant effect of OBT-A has been suggested in different conditions [55,56]. In a recent study [57], a clinically meaningful reduction of depression and anxiety symptoms, as measured using self-reported questionnaires, emerged, even in the case of OBT-A non-responders in terms of reduction of headache days. The potential mechanism of antidepressant action of OBT-A remains unknown. In the case of patients with CM, the relief from the symptoms burden might induce, in turn, a reduction of psychiatric symptoms. However, an OBT-A antidepressant effect per se has been hypothesized [57].

Interestingly, lower depression symptoms emerged as predictors of OBT-A responders [58,59] and only patients without negative emotional states reported improved sleep quality as a benefit of OBT-A [53]. Certain psychological features and personality traits (i.e., dependent trait) have been associated with OBT-A non-response in patients with CM [60]. In our previous study, we reported that specific coping strategies interfere with analgesic consumption and they are associated with different acute medication consumptions behaviors in patients with CM undergoing OBT-A [61].

To sum up, the literature supports the importance to explore emotional distress and psychological profiles in patients undergoing OBT-A and in other CM management phases. A more personalized treatment approach, combining both pharmacological and non-pharmacological treatment options, is warranted, especially in Italy [30].

In line with this background, the study aims to (i) describe psychological distress, self-efficacy, coping strategies, perceived social support, psychological flexibility, and attitude towards psychological support in a cohort of patients with CM attending OBT-A or WT; (ii) to characterize and compare psychological distress and characteristics of patients undergoing OBT-A with patients undergoing WT; and, (iii) to evaluate the factors influencing psychological distress, also considering the CM management phase.

## 2. Results

### 2.1. Socio-Demographic and Clinical Characteristics

According to the selection criteria, 111 patients were eligible for inclusion in the two centers. Thirteen patients receiving OBT-A and 11 attending WT declined to participate or did not sign informed consent. Patients undergoing WT were all diagnosed with MOH, while seven patients (17.5%) among those undergoing OBT-A reported medication overuse based on the screening of the drugs consumption diary in the three months before the administration of the questionnaires. Moreover, half of these patients reported a history of at least one WT.

Table 1 reports socio-demographic and clinical characteristics of the 87 participants. The two groups were homogeneous for age, gender, educational level, and civil status. Female mainly composed the sample. Most patients reported a severe migraine-related disability. However, patients with OBT-A reported a lower percentage of severe migraine disability (80%) and a higher percentage of mild-moderate one (20%) than patients attending WT (93.5% and 6.5%, respectively). The pain intensity was rated at almost seven in both groups. Patients declared to have headaches for more than half days of the previous three months on average; headache frequency was significantly higher for patients attending WT (*p* < 0.01).

### 2.2. Psychosocial Characteristics and Psychological Distress in CM Sample

Table 2 reports psychological distress and characteristics. For the whole sample, 38% of patients reported a General Severity Index (GSI-T score) equal to or higher than 55. The majority of included patients presented coping strategies mean values that were comparable to those of the general population included in the validation study of the Coping Orientation to the Problems Experienced (COPE) [62], with higher use of social support (validation study mean = 27.7; DS = 8.4; *t* = 3.54 *p* < 0.01) and of avoidance strategies (validation study mean = 23.5; DS = 5.1; *t* = 2.55 *p* = 0.01). The mean values of the perceived social support resulted in line with the score that was reported in the Italian validation study of the Multidimensional Scale of Perceived Social Support (MSPSS) [63], with a slightly higher value for social support by a specific person (validation study mean = 5.6; DS = 1.4; *t* = 4.01 *p* < 0.01). The mean score of Acceptance and Action Questionnaire II (AAQ-II) shows a higher level of psychological inflexibility (validation study mean = 20.1; DS = 7.3; *t* = 2.6; *p* = 0.01) [64]. Equal mean values of perceived self-efficacy resulted when compared with D’Amico study (CM patients with MOH mean = 2.9, DS = 0.5, *t* = 1.55, *p* = 0.12) [45].

### 2.3. Comparison of Psychosocial Characteristics and Psychological Distress: OBT-A versus WT

As reported in Table 2, 25% of patients attending the OBT-A reported a psychological distress of at least moderate-severe level. Only 5% of patients attending OBT-A resulted over the clinical cut-off (GSI-T > 65) against the 27% of patients attending WT. Similarly, the OBT-A group reported lower Positive Symptoms and Positive Symptoms Distress than the group undergoing WT.

No significant differences in coping strategies, perceived self–efficacy, and perceived social support were reported between groups. Conversely, patients attending OBT-A reported a significant lower psychological inflexibility than WT, for whom the level of psychological inflexibility/experiential avoidance also resulted in being higher than the one reported in the validation study of the Acceptance and Action Questionnaire II (AAQ-II) (*t* = 0.73 *p* = 0.46 and *t* = 4.24 *p* < 0.01) [64].

### 2.4. Psychosocial Factors Influencing Psychological Distress

Pearson correlation coefficients showed that the psychological distress, as measured by GSI-T scores, was strongly associated with psychological inflexibility and avoidance coping strategy (*r* = 0.7, *p* < 0.01 and *r* = 0.6; *p* < 0.01 respectively), followed by weak links with migraine frequency, perceived self-efficacy, and perceived friend support (*r* = 0.3; *p* < 0.05 for MIDAS-C, MSPSS-FR, and GSE).

A more in-depth subgroup analysis on GSI-T scores, distinct for patients with CM undergoing OBT-A and WT, underlined a different intensity between distress and perceived self-efficacy (*r* = −0.5, *p* < 0.01 and *r* = −0.2, ns, respectively).

Table 3 reports the regression model that was performed over GSI-T scores, jointly considering the variables that were deemed significant in the bivariate analysis.

The resulting model explained 51% of GSI-T scores variation. Although CM management phase predicted psychological distress (i.e., attending the WT predicted higher psychological distress, confirming the higher level of psychological distress in this group), perceived social support by friends, self-efficacy, and coping avoidance strategies all remained relevant independent psychological predictors.

### 2.5. Psychological Support Received, Awareness of Psychological Problems and Opinion on the Utility of Psychological Strategies for CM Management

As described in Table 4, 28% of patients with CM declared to have suffered, or currently suffer, of a psychological problem, and 36% to have received or have been receiving psychological support for distress linked to migraine.

Seventy-nine per cent of responders considered it useful to receive psychological strategies, with interest for psychological strategies reaching 86% for the patients attending the WT, although no significant difference emerged between the two groups.

Regardless of the level of psychological distress, patients showed interest in receiving psychological support in dealing with migraine distress. Only four patients (two in each sub-sample) among those who declared not to be interested in receiving psychological strategies reported equal or higher values than the GSI-T 55 score.

## 3. Discussion

This study explored the emotional distress and psychological profiles of patients with CM attending two different programs of CM management: a withdrawal program for patients with MOH in a hospital setting and a prophylactic program with periodical injections of OnabotulinumtoxinA in an outpatient setting.

Patients attending OBT-A presented lower frequency of migraine attacks. However, the severity of the migraine-linked disability and the socio-demographic characteristics of the two groups resulted in being comparable. A high level of psychological distress emerged, with around one-third of the whole sample presenting at least moderate-severe psychological distress. Among those patients, around 17% reported very severe psychological distress over the clinical cut-off, suggesting a referral for a more in-depth assessment of psychopathology. Psychological distress resulted in being particularly high for patients undergoing WT, half of whom reported at least moderate-severe emotional distress. These results confirmed previous researches, which highlighted multiple psychological comorbidities in patients with MOH [21]. Saper and Lake [65] suggested the presence of one sub-group of patients with MOH with a psychological dependence component, needing specific multidisciplinary approach before and after withdrawal [66]. More recently, higher depression scores resulted in being associated with a negative outcome for withdrawal [14] and baseline self-reported depression resulted associated with the relapse in MOH and need for another WT at one year [67].

Although the percentage of patients with psychological distress over the clinical cut-off was low, one out of four patients attending OBT-A presented at least a moderate-severe level of psychological distress. Therefore, identifying patients presenting psychological vulnerabilities is also essential during the OBT-A. On the one hand, it allows identifying patients with a poor predictor of OBT-A response, due to the presence of depression and psychological distress [53,58,59,61]. On the other hand, patients give more value, as assessed by clinicians, to the quality of life among OBT-A outcomes [68].

The reduced level of psychological distress within the OBT-A group might be partially explained by previous literature. Patients undergoing WT reported very high frequent migraine attacks when compared with the OBT-A group. Indeed, psychological distress might be associated with migraine frequency when comparing CM versus EM or other categories of attack frequencies [16,17,24,25]. The higher level of psychological distress for patients undergoing WT might be associated with the diagnosis of MOH. However, few patients undergoing OBT-A reported medication overuse likewise. Previous studies reported that a reduced level of distress could be a beneficial effect of the OBT-A [51,52,57]. The fact that we explored this potential association on a cross-sectional level without assessing changes in clinical outcomes and psychological distress during OBT-A treatment does not allow for us to evaluate whether the prophylactic treatment might influence clinical outcomes. The longitudinal monitoring of the psychological distress in patients undergoing OBT-A might allow investigating OBT-A clinical effect in reducing depression, anxiety, and other psychological symptoms.

Interestingly, patients attending OBT-A presented lower psychological inflexibility, similarly to the general population scores: a psychological dimension related to a worse level of mental health as well as the development and maintenance of higher levels of psychopathology [64,69]. In the migraine field, the association between higher headache-related acceptance and lower level of depression and anxiety has been suggested by Seng [34]. Coherently, psychological inflexibility resulted in being strongly associated with headache-related disability in a previous study, including patients with migraine (mainly EC) [44]. Conversely, MOH patients undergoing WT reported a high tendency to experiential avoidance, and they also reported higher psychological inflexibility than the population of the AAQ-II validation study, suggesting acceptance and values-based action as relevant psychological dimensions to be fostered through appropriate psychological strategies for patients with MOH. Recently, Acceptance and Commitment Therapy (ACT) showed promising results in reducing adverse impact in chronic pain conditions, including patients with migraine [32,70,71,72,73].

As regards coping strategies, a higher use of avoidance strategies was confirmed in the whole sample, and avoidance resulted in being the only coping strategy associated to psychological distress, suggesting the relevance of modifying this dysfunctional way to cope with stress when helping patients, as already discussed [46]. Looking to the other coping strategies, our sample showed results that were comparable to those reported in the validation study of the COPE [62], even with a higher use of social support strategies. D’Amico [45] suggested the possible involvement of supportive relationships in the prevention of MOH relapse. Perceived social support has been recognized as having a considerable positive influence on mental health. However, the effect might vary, depending on the source of the support [74]. Our study supports these results and shows that, in patients with CM, higher perceived support by friends is associated with lower psychological distress. A slightly higher value for perceived social support by “a specific person” compared to the general population emerged. Qualitative studies have reported that patients with CM consider a close relationship helpful in the management of CM, even though relationships and perception of social support can be “strained”, with others potentially being “overprotective or undermining” [4]. Moreover, as in other chronic conditions, migraine might affect the wellbeing of supporting people and the interpersonal dynamic thus affecting the patient’s wellbeing [3].

Despite the different level of headache frequency, self-efficacy resulted in being similar in both groups. Perceived self-efficacy has been considered a key factor in migraine management, adaptation to pain, prevention and management at the onset or in the course of headache attacks and resulted in being associated with headache-related disability [45,47,48,61]. Interestingly, only for patients undergoing OBT-A low level of perceived self-efficacy resulted in being associated with higher psychological distress.

Finally, as regards our third aim, higher use of avoidance strategies and lower self-efficacy and friends support resulted in being related to higher psychological distress, beyond the different severity of CM in the two groups. Tailored psychological interventions dedicated to these patients might contribute to reduce emotional distress and psychological comorbidities and foster a more functional psychological management of stressors of life, including the prevention and management of headache attacks; all aspects that might influence the migraine-linked disability [34,45,75] and long-term prognosis of CM, by influencing the threshold for the generation of migraine attacks [11].

As regards some practical implications of our findings, our study reinforced the need to adopt a “patient-centered care” focus based on an integrated and multidisciplinary clinical assessment [29]. Validated psychological scales should be used for preliminary screening of psychological complaints in patients with CM either in the presence of medication overuse or during the prophylactic treatment using OBT-A. With this perspective, periodical OBT-A injections should be associated with periodical psychological assessment other than the other clinical outcomes (e.g., disability scales, headache diary). The outpatient OBT-A setting is relevant for the integrated psychosocial assessment and interventions within a multidisciplinary perspective [76] involving different units. The outpatient context offers opportunities, such as socialization and engagement, in valued actions at work, at home or in the community, which represents a precise starting point for psychological and behavioral support.

Of note, the perspective of patients regarding psychological needs and support has been scarcely explored in the context of CM [4,42]. In our study, most of the patients evaluated the utility of psychological strategies for CM management positively, independently by the group, and even with low psychological distress. Further studies are warranted to explore the patient’s motivation and opinions on psychological interventions for CM, using focus groups or interviews. The patients definite answer on receiving psychological strategies is a positive starting point to make complementary behavioral interventions available. In the literature, the recognition of the role of psychosocial aspects in migraine is increasing, with a definite interest in psychological approaches to be integrated with pharmacological and medical ones and a growing interest in, and utilization of, complementary and integrative strategies, such as mindfulness-based interventions [37,38,73]. However, a gap between research on the relevance and effectiveness of behavioral treatment interventions and the clinical practice remains [37,77,78].

### Strengths and Limitations

Having characterized the specific psychological aspects of different sub-populations of CM patients attending different CM management phases, both in terms of psychological distress and psychological profiles, extends previous findings and provides insights into the psychosocial needs of a less studied group of patients with CM undergoing OBT-A.

The utilization of a self- reported instrument, like the SCL-90r, represents a strength of the study, because it overcomes the only focus on depression and anxiety symptoms. Other psychological symptoms other than depression and anxiety can be present and contribute to the overall distress of the patient [19]. Moreover, this multidimensional scale of psychological distress can be useful for the initial screening (but not for the diagnosis) of potential candidates for additional psychological assessment.

The over-representation of patients with very severe CM does not allow generalizing findings to other contexts of care, or patients with migraine or CM managed in primary care, or not seeking medical treatment. Finally, the study could not be balanced for gender since the results are mainly based on a female cohort. Having most female patients among the sample resulted in line with the female over-representation in this disorder [79]. However, social role expectations and coping styles differ between genders potentially affecting the experience of headache and pain [80]. The two treatment groups differed in the severity of the disease (frequency of attacks). The link between frequency and distress was not explored in the study, thus representing a major weakness.

Further studies are needed, allowing for a better characterization of this population. Seven patients undergoing OBT-A reported medication overuse according to the guidelines, and three of them reduced the consumption of drugs in the three subsequent months below the level that was considered for MOH diagnosis. Future studies should compare psychological distress and profiles between CM patients with and without overuse in a larger sample of patients treated with OBT-A.

The cross-sectional nature of the current study did not allow us to assess changes in psychological distress and factors over time, as previously discussed [61], or to verify whether OBT-A is influencing distress directly or due to CM relief. These aspects should be further explored in a longitudinal study evaluating psychological distress and factors from the baseline over OBT-A follow-up periodical injections. Moreover, we were not able to test whether patients reported low self-efficacy, psychological flexibility, perceived social support because of the psychological distress, or vice versa [45]. As regards this causality hypothesis, from a clinical practice point of view, these psychological variables might intersect with the psychological distress and contribute to its sustaining, creating a vicious circle that maintains the suffering over time. As previously discussed, a multidisciplinary approach taking into consideration all of these relevant bio-psycho-social aspects is necessary in order to detect the specific role of these factors during each phase of CM management and introduce tailored intervention in order to enhance the effect of specific CM treatment.

## 4. Conclusions

The current study shows the presence of different levels of psychological distress and psychological profiles in patients with CM undergoing OBT-A or WT. The perceived level of psychological distress is relevant in patients with medication overuse attending a withdrawal program, but it could not be excluded, even in patients receiving OBT-A with lower symptoms severity. To conclude, it is worth noting that most patients included in the study were interested in receiving psychological strategies for the management of migraine. Psychological assessment and interventions might enhance the therapeutic intervention in patients with CM within a bio-psycho-social framework.

## 5. Materials and Methods

### 5.1. Study Design and Setting

A cross-sectional observational cohort study was performed from February 2018 to April 2019. The study is part of the project “EXploring PsychoLOgical needs of patients with chronic pain attending neuroREhabilitation services” (EXPLORE) [61]. Ethical approval to conduct the study was provided by the Ethics Committee of the Verona and Rovigo Province in Italy (approval date: 17 January 2018, 1630CESC, Prog. n. 14112; 27 February 2018 Prog. 1667CESC, Prog. n. 11576).

The study was conducted through the strict collaboration among the UOC Neurorehabilitation and the Unit of Clinical Psychology (University hospital, AOUI Verona, Verona, Italy) and the Neurological Unit and the Clinical Psychological Service of the Scientific Institute for Research Hospitalisation and Health Care (IRCSS) Sacro Cuore (Don Calabria Hospital, Negrar di Valpolicella, Italy). Since 2017, these clinical centers collaborate for the management of CM patients in an interdisciplinary context (neurologists, rehabilitation physicians, psychologists, physical therapists) to implement a pathway of care in order to address the multiple factors (i.e., therapeutic exercise, myofascial dysfunction) that may contribute to decreased migraine severity [31,61].

The patient refers to the specialist in Neurology at the IRCSS to define the diagnosis and plan the pharmacological treatment according to the CM symptoms severity and the patients’ needs [50]. The OBT-A or WT decision was based according to the diagnosis and the clinical expertise of the single neurologist (F.M.). In the presence of MOH (10 or more days/month of triptan/opioid intake or 15 or more days/month of simple analgesics or combination for more than three months), the patient was admitted to the Neurologic Unit for a 10-day hospitalization WT program [81]. Drug detoxification was performed as an in-patient program and consisted of advice, steroids, anxiolytic drugs, fluid replacement, and antiemetics preventive treatment [82]. Patients who have failed at least two to three other migraine prophylactics were referred at the Neurorehabilitation Unit (AOUI Verona, Verona, Italy) as an outpatient for the OBT-A prophylaxis [50]. It consists of OBT-A injection managed by the same physician, during periodical outpatient visits every 12 weeks, according to the international protocol [31,61].

### 5.2. Participants and Procedures

In the outpatient center, the patients were approached after the follow-up periodical outpatient visit for OBT-A, while in the IRCCS of Negrar di Valpolicella during the hospitalization for the WT program. Patients were assessed for eligibility according to the following inclusion criteria: fulfilment of ICHD-III beta criteria for CM [10], as diagnosed by a neurologist (i.e., ≥15 headache days per month, ≥8 of which are migraine days); age between 18 and 80; and, the ability to understand the purpose of the study as evaluated by researchers approaching the patients. Patients who were already evaluated for eligibility in one center were not considered to be eligible for recruitment in the other one. After signing the informed written consent, participants entered the study and filled a series of self-reported questionnaires.

### 5.3. Measures

Gender, age, educational level, civil status and work condition were collected through an ad hoc form.

The impact of migraine on daily functioning over the past three months was assessed using the Migraine Disability Assessment (MIDAS) Questionnaire [83]. The MIDAS total score—the sum of five items examining the disability in school/work, household work, and family/social/leisure activities—were categorized in four categories: I (MIDAS score 0–5), II (6–10), III (11–20), and IV (>20), with grade IV corresponding to high disability level and grade I, II, III being collapsed in a mild-moderate level. Two additional questions (MIDAS-C and MIDAS-D) asked patients to evaluate the number of days with headache (frequency of headache in the last three months) and the painfulness of headache attacks (from 0 to 10).

Table 5 summarizes the main psychological variables that were explored in the study and describes the self-reported questionnaires used. The scales have been previously used in the context of migraine or clinical populations [41,45,61,84,85].

Finally, patients were asked to answer the following dichotomous questions: “Do you suffer or have suffered in the past of a psychological problem?”, “Have you received, or are you receiving psychological support for distress connected to migraine?”, and “Do you think that it might be useful to receive suggestions on how to psychologically deal with migraine management?” This last question aimed at exploring attitudes toward psychological support.

### 5.4. Statistical Analysis

Psychological scale scores with more than 10% of item non-response were not calculated, so that a pairwise deletion approach conducted the analysis. When less than 10% of responses were missing, the averages of the available item responses were performed as the imputation method.

Descriptive statistics are presented as percentages, means, and standard deviations. Psychological characteristics of the sample have been compared with values reported in the validation study of the scale in order to better understand the psychological needs of the CM sample in comparison with the general population, by using Student t-test for independent samples. T-test or chi-square test were applied for comparing the socio-demographic, clinical, and psychological variables between the two CM groups.

Pearson’s correlation coefficient was calculated to explore the relationships between psychological variables. Multiple linear regression analyses were applied in order to explore the relationships among the Global Severity Index (GSI)-T score, as the main outcome variable, socio-demographic and the other psychological characteristics, as the explanatory ones. Regarding the choice of the final model, a parsimonious criterion was applied to take the dimension of the sample into account.

The multi-center structure of the sample was accounted for in the regression models introducing the center as a stratification variable, in order to consider the differences between OBT-A and WT group and explain potential undetected features.

All of the analyses were performed using STATA 15.1 (StataCorp. 2017, College Station, TX, USA).

## Figures and Tables

**Table 1:** Socio-Demographic and Clinical Characteristics of Patients distinguished by Treatment Group.

	All Patients	OBT-A	Withdrawal Treatment	
Socio-Demographic and Clinical Characteristics	N (%)	N (%)	N (%)	*t*-Test or Chi2
Participants	87	40 (46.0)	47 (54.0)	
Age, *mean* (*SD*)	46.2 (12.8)	46.8 (13.9)	45.7 (11.9)	−0.4
Gender, *female*	76 (87.4)	37 (92.5)	39 (83.0)	1.8
Education °				
Secondary School	23 (27.4)	11 (28.2)	12 (26.7)	
High School	34 (40.5)	13 (33.3)	21 (46.7)	
Degree or Post Degree	27 (32.1)	15 (38.5)	12 (26.7)	1.8
Civil Status °				
Single	24 (28.6)	12 (30.8)	12 (26.7)	
Married or Unmarried Partner	51 (60.7)	24 (61.5)	27 (60.0)	
Separated/Divorced/Widowed	9 (10.7)	3 (7.7)	6 (13.3)	0.8
Current Working Condition ^§^				
Not employed	28 (32.9)	11 (28.2)	17 (37.0)	
Employed	57 (67.1)	28 (71.8)	29 (63.0)	0.7
Headache-related Disability (MIDAS)				
Low to Moderate Disability (score < 21) °°	11 (12.8)	8 (20.0)	3 (6.5)	
Severe disability (score ≥ 21)	75 (87.2)	32 (80.0)	43 (93.5)	3.5
MIDAS-C Headache Frequency, *mean* (*SD*) °°	54.0 (30.3)	38.0 (23.5)	67.9 (28.8)	−5.2 **
MIDAS-D Pain Intensity, *mean* (*SD*) ^§§^	6.7 (2.0)	6.3 (1.6)	7.0 (2.2)	−1.6

Legend: OBT-A, OnabotulinumtoxinA Prophylactic Treatment; N, number; %, percentage; SD, standard deviation; MIDAS, Migraine Disability Assessment; ** *p* < 0.01; ° 3 missing values; ^§^ 2 missing values; °° 1 missing value; ^§§^ 6 missing values.

**Table 2:** Psychological Distress and Psychological Traits of Patients with chronic migraine (CM) distinguished by Treatment Group.

	All Patients	OBT-A	Withdrawal Treatment	
Psychological Outcomes	N (%)	N (%)	N (%)	Chi2
Psychological Distress (SCL-90-R) ^§^				
General Severity Index (GSI)				8.0 *
T ≤ 54	53 (62.4)	30 (75.0)	23 (51.1)	
T 55–64	18 (21.2)	8 (20.0)	10 (22.2)	
T ≥ 65	14 (16.5)	2 (5.0)	12 (26.7)	
Positive Symptom Distress Index				7.4 *
T < 54	63 (74.1)	35 (87.5)	28 (62.2)	
T 55–64	15 (17.7)	4 (10.0)	11 (24.4)	
T ≥ 65	7 (8.2)	1 (2.5)	6 (13.3)	
Positive Symptom Total				11.4 **
T < 54	36 (42.4)	22 (55.0)	14 (31.1)	
T 55–64	29 (34.1)	15 (37.5)	14 (31.1)	
T ≥ 65	20 (23.5)	3 (7.5)	17 (37.8)	
	**Mean (SD)**	**Mean (SD)**	**Mean (SD)**	***t*-test**
Perceived Self-Efficacy (GSE) °°	2.8 (0.5)	2.8 (0.5)	2.8 (0.5)	0.4
Coping Strategies (COPE)				
Social Support (COPE-SS) °	30.6 (6.7)	29.9 (6.8)	31.1 (6.6)	−0.8
Avoidance (COPE-AS) ^§^	25.3 (6.2)	24.1 (5.1)	26.5 (6.9)	−1.8
Positive Attitude (COPE-PA) ^§^	30.8 (6.2)	29.9 (6.2)	31.7 (6.1)	−1.3
Problem-Oriented (COPE-PO) ^§^	31.3 (5.7)	30.8 (5.7)	31.8 (5.7)	−0.8
Turning to Religion (COPE-TR) °	22.0 (4.8)	22.3 (5.0)	21.8 (4.6)	0.5
Psychological Inflexibility (AAQ-II) °°	23.3 (10.5)	19.2 (8.9)	26.8 (10.5)	−3.6 **
Perceived Social Support (MSPSS) °°				
By Significant Others (MSPSS-O)	6.0 (1.3)	5.9 (1.3)	6.2 (1.3)	−0.8
By Family (MSPSS-FA)	5.8 (1.6)	5.7 (1.7)	5.8 (1.5)	−0.3
By F0riends (MSPSS-FR)	4.8 (1.8)	4.5 (2.0)	5.1 (1.6)	−1.6

Legend: OBT-A, OnabotulinumtoxinA Prophylactic Treatment; N, number; %, percentage; SD, standard deviation; SCL-90-R, The Symptom Check List-90-R; GSE, The General Self-Efficacy Scale; COPE, Coping Orientation to the Problems Experienced; SS, Social Support; AS, Avoidance Strategies; PA, Positive Attitude; PO, Problem Oriented; TR, Turning to religion; AAQ-II, The Acceptance and Action Questionnaire II; MSPSS, Multidimensional Scale of Perceived Social Support; O, by Significant Other; FA, by Family; FR, by friends; * *p* < 0.05; ** *p* < 0.01; °° 1 missing value; ^§^ 2 missing values; ° 3 missing values.

**Table 3:** Psychological Factors influencing Psychological Distress.

	Psychological Distress (GSI—T Score)		
	*R*^2^ = 0.51		
Explanatory Variables	Coef.	[95% Conf. Interval]	
Perceived Social Support by Friends (MSPSS-FR)	−1.4	−2.4	−0.4
Avoidance Coping Strategies (COPE-AS)	0.8	0.5	1.1
Perceived Self-Efficacy (GSE)	−5.3	−9.0	−1.6
Withdrawal Treatment (ref. OBT-A)	6.6	3.1	10.1
(Constant)	51.0	36.6	65.5

Legend: GSI—T score, Global Severity Index using a T-score approach; OBT-A, OnabotulinumtoxinA Prophylactic Treatment; GSE, The General Self-Efficacy Scale; COPE, Coping Orientation to the Problems Experienced; AS, Avoidance Strategies; MSPSS, Multidimensional Scale of Perceived Social Support; FR, by friends.

**Table 4:** Attitude towards Psychological Support in patients with CM, distinguished by Treatment Group.

	All Patients	OBT-A	Withdrawal Treatment	
Items	N (%)	N (%)	N (%)	Chi2
Suffering or having suffering of a psychological problem (self-reported) °°—*Yes*	24 (27.9)	11 (28.2)	13 (27.7)	0.0
Receiving or having received psychological support °°—*Yes*	31 (36.1)	13 (33.3)	18 (38.3)	0.2
Interest for receiving psychological strategies ^§§^—*Yes*	64 (79.0)	28 (71.8)	36 (85.7)	2.4

Legend: OBT-A, OnabotulinumtoxinA Prophylactic treatment; N, number; %, percentage; SD, standard deviation; °° 1 missing value; ^§§^ 6 missing values.

**Table 5:** List of Psychological Questionnaires adopted in the study.

Variables	Psychological Questionnaire	Main Characteristics and Scores
Psychological Distress	The Symptom Check List-90-R [86]	90 items rated on a 5-point Likert scale (from “not at all” to “extremely”). 3 global indexes have been calculated: the global severity index (GSI)—an overall measure of psychological distress; the positive symptom total (PST)—number of items scored above zero, reflects the symptoms’ breadth; the positive symptom distress index (PSDI)—the average level of distress for the self-reported symptoms, indicating the perceived intensity of symptoms. Data have been reported using a T-score approach: GSI or PST or PSDI, T scores below the cut off of 55 indicate a level of the specific index similar to the mean of the general population; T scores between 55 and 64 indicate a moderate-severe level of psychological distress (GSI), a moderate-high a number of symptoms (PST) and a moderate-high intensity of symptoms (PSDI) respectively; at T scores higher than or equal to 65, the level of GSI becomes very severe and the number of symptoms or their intensity is considered high [87,88]
Coping Strategies	Coping Orientation to the Problems Experienced (COPE) [62,89,90]	60 items rated on a 4-point scale (from ‘‘usually do not do this at all’’ to ‘‘usually do this a lot’’). 5 essentially, independent coping strategies have been calculated: social support (COPE-SS)—seeking social support to obtain advice, information or sympathy from others and express emotions; avoidance strategies (COPE-AS)—the attempts to avoid dealing with either the problem or the associated emotions, including denial; behavioural and mental disengagement; helplessness attitude and substance utilization to reduce distress; positive attitude (COPE-PA)—the attitude of acceptance; containment; positive reinterpretation of events and restraint-coping; problem-solving orientation (COPE-PO)—the active involvement in dealing with the source of stress through taking steps to eliminate the problem, planning and suppression of competing activities; turning to religion (COPE-TR)—the use of faith for support and the absence of humour.
Perceived self-efficacy	The General Self-Efficacy Scale (GSE) [91,92,93]	10 items rated on a 4-point scale (from “not at all true” to “exactly true”). Higher scores correspond to higher levels of perceived self-efficacy.
Psychological Flexibility	The Acceptance and Action Questionnaire II (AAQ-II) [64,94]	7 items on a 7-point scale (from “never true” to “always true”). Higher scores correspond to higher levels of psychological inflexibility, which is conceived experiential avoidance. “The phenomenon that occurs when a person is unwilling to remain in contact with particular private experiences (e.g., bodily sensations, emotions, thoughts, memories, images, behavioural predispositions) and takes steps to alter the form or frequency of these experiences or the contexts that occasion them, even when these forms of avoidance cause behavioural harm” [95].
Perceived Social Support	Multidimensional Scale of Perceived Social Support (MSPSS) [63,96]	12 items rated on a 7-point scale (from “full disagreement” to “full agreement”). 3 mean perceived support scores have been calculated: perceived social support by family (MSPSS-FA), by friends (MSPSS-FR), by significant others (MSPSS-O).
